# National Dental Association, Niagara Falls, August 1-4, 1899

**Published:** 1899-12

**Authors:** J. M. Walker


### PROCEEDINGS.

### National Dental Association, Niagara Falls, August 1-4, 1899.

Reported for the DENTAL REGISTER by Mrs. J. M. Walker.

(*Continued from November No., page* 514.)

### CEMENTS.

BY DR. E. J. WEDELSTAEDT, ST. PAUL, MINN.

The cements having been in use for the last thirty years, it seems strange that it has not been recognized that the majority of those in use are very readily penetrated by moisture. If each one, the next time he uses cement, will mix a little more than is needed, and place the residue in a bottle of ink for from twenty-four to forty-eight hours, he can easily ascertain the extent of penetration. If he finds *one* that is not penetrated, that is the one to stick to. Where ink can penetrate, saliva can penetrate also, and where saliva can penetrate, micro-organisms can go.

Dr. Wedelstaedt then produced a number of glass tubes containing aniline solutions, in which, on July 12th, there had been placed mixes of different cements, those in one tube having been covered with sandarac varnish; in another with hot melted paraffin, the third having had no preparation.

Dr. C. N. Johnson was requested to open the tubes and report the condition of the cement masses, with the result that the cements tested were pronounced by Dr. Johnson not to be fit for use in the teeth, all of the cylinders showing more or less penetration and discoloration by the aniline solution.

Dr. Wedelstaedt said that other experiments had shown that there was also both expansion and contraction to be overcome in the different cements on the market, one expanding 2 m. beyond the cavity, another contracting to the extent of a line running completely around the filling. Microscopic examination of the different powders also revealed bits of wood, cotton, hair, etc.

Age has a remarkable effect upon the resisting power of the cements as tested by the dynamometer. In one case a cement which carried but 30 or 35 pounds at the end of 24 hours carried 400 pounds after 96 hours. He concluded his paper by saying that when we demand cements that will neither shrink nor expand, and that moisture will not penetrate, we will get them, and it will be the better for our patients the sooner it is done.

### SOME PHASES OF THE CEMENT QUESTION.

BY DR. WM. V. B. AMES, CHICAGO.

In the consideration of the physical properties of filling materials, the cements should have a share of attention. Among the questions to be dealt with are the crystallization of cement liquids; shrinkage and expansion; the coarse or fine powders; arsenical contamination of cement powders, etc. The crystals which separate from the fluid, whether held in suspension, or adhering to the sides of the bottle, or settling to the bottom in a mass, were originally part of the solution, evenly distributed throughout, so that their separation and loss must impair the virtues of the original formula. The crystals may, however, usually be liquefied by heat and restored to the fluid, or they may be rubbed down and liquefied in the mix of the cement.

Dynamometer tests show an increase of edge strength up to a point somewhat short of impalpability in the powder; we may expect greater strength of contour when a slight grit is desirable, though the granular state may detract from the ability of the mass to withstand long-continued attrition.

As to shrinkage and expansion, when the phosphoric acid has been modified by alkaline phosphates only, the basic phosphate which is formed is of a friable nature and exerts no special force in drawing together the granules, making evident shrinkage at the periphery. But when the acid has been modified by non-alkaline phosphates the basic phosphate formed agglutinates, the zinc oxide granules, drawing them toward a center and giving a tendency to a diminution of volume during crystallization, depending upon lack of water—of crystallization. This, if present, would give too rapid setting, but which if added to the crystallizing mass will be taken up and given the difference between shrinkage and expansion.

When the mass is allowed to harden in a tube in the dry state, wholly unlike conditions exist to those present when the cement is used in the mouth. There is a disadvantage rather than otherwise, in long-continued protection from the saliva, in the use of the cements in which the phosphoric acid is modified by non-alkaline phosphates.

Arsenical contamination of the cement powders is easily demonstrated, but the zinc-arsenic compound is inert and wholly devoid of poisonous properties *per se*, and may be wholly ignored.

DISCUSSION.

In the discussion of the subject, DR. G. V. BLACK said that it is greatly to be desired that the physical properties of the cements should be investigated, as it has not yet been shown that we have a reliable cement to-day, one that will do what cement fillings are expected to do. Unless each batch is tested we do not know whether it will shrink or expand. From observations made in his own laboratory, he found that in a well-prepared cavity there was sufficient shrinkage in an hour or so to let arsenic out on to the gums if it had been used as covering for an arsenical application.

DR. J. D. PATTERSON said he did not see how it could be possible for Dr. Wedelstaedt to make a proper mix in such large masses.

DR. WEDELSTAEDT replied that each of the "fillings" represented the entire mass, and the mixing was thoroughly done.

DR. CROUSE hoped more men would take up this work so that we would eventually have a cement that would not shrink, that would not be porous and from under which arsenic could not escape.

Dr. Ames said that from his own experiments in this direction he had reason to believe that the tendency to shrinkage would yet be overcome, and that we would eventually have a cement that will be impervious to moisture.

### SUSCEPTIBILITY AND IMMUNITY TO DENTAL CARIES.

BY DR. G. V. BLACK, CHICAGO.

Caries is, and has been, so common that it has come to be considered that practically all are afflicted. And yet there is a sufficient number of exceptions for all who have been long in practice to have known some persons who have been wholly immune during a long lifetime. This has been erroneously attributed to a superior quality of the teeth; but caries is not dependent upon the teeth, which are all nearly the same in their calcification, though in their physical structure there are wide differences. Developmental grooves may be imperfectly closed, having fissures, pits and openings; the dentin may have interglobular spaces, but these and other physical imperfections are not *causes* of decay; they only give opportunity for the action of the causes when present and active. In caries the teeth are acted upon; the agents acting to produce decay are outside of the teeth and must be found in their environment. Caries has its beginning only when the conditions of the oral secretions are such that the tissue organisms causing decay form gelatinous plaques by which they are glued to the surface of the teeth. So long as they have no protection from the dissipation of the acids they form they can not produce caries. The organisms grow in every mouth, but the formation of the gelatinous plaque does not occur in every mouth, and this seems to depend upon something in the saliva the nature of which is yet unknown. Researches for the factors which predispose to decay should be sought, therefore, not in the teeth themselves, which are the most unchanging of the tissues of the body, but in the surroundings of the teeth, in the oral fluids, and in the bodily conditions which give character to the secretions, the latter being affected by the slightest causes.

Susceptibility to caries of the teeth is influenced by heredity, by age, and by fluctuations of bodily conditions. The hereditary predisposition to caries is of the first importance, as in children living under conditions similar to those of the parents in their childhood, the susceptibility to caries will be very similar in a majority of cases. The hereditary predisposition disappears with the coming of adult age. The effect of early radical treatment by filling is that the person becomes immune at the time of life at which caries would be making its worst ravages if early filling had not been done. This is partly due to better care, the better care being due to the better conditions which made the better care possible. Full and natural use of the teeth in the mastication of food, and the abrasive action of the food in cleansing the teeth, are important elements in bringing about an early abatement of susceptibility to caries. Through reaction a better condition of the secretions is produced. For children, therefore, the most perfect operations are demanded in order to maintain the full, free and efficient use of the teeth. The practice of making permanent fillings for children has been severely criticised, but there are no good grounds for this condemnation. The fact that the pulp occupies larger space than in adult teeth simply demands corresponding care and judgment. Judicious management is required, and the endurance of a child should never be severely taxed; the nervous system should be looked to with the greatest care. When permanent operations can not be made, the case must be tided along until better conditions obtain.

Local immunity is due, not to differences in the structure of the teeth, but to environment, certain localities favoring the protection of microbic plaques. Without careful consideration of these questions clinical experience will lead to conclusions very wide from the facts.

### MANAGEMENT OF CHILDREN'S TEETH.

BY DR. C. N. JOHNSON, CHICAGO.

In the management of the deciduous teeth, palliative work, keeping the patient comfortable for a few years, is usually the object. With the permanent teeth—those which appear early in life—the aim should be given the greatest permanency to the operations, with the thought ever in mind that the highest possibilities in dental art involves saving these organs for a life time.

The assumption that the deciduous teeth may be neglected, however, because they will eventually be lost, should be combated at every opportunity. This is not only because of the possible suffering, and the injury to health resulting from lack of mastication, and from the presence of diseased and abscessed teeth, but especially is the question of acquired habits which may conduce to permanent injury. The habit of bolting the food unmasticated, and therefore unfit for service, is one that often clings through life. Effective mastication is a weighty factor in the health and longevity of the individual, and it is all-important that the teeth of children shall be kept in such condition as shall conduce to habits of thorough mastication. If a pulp is exposed in a deciduous tooth, syringe well with tepid water, and remove anything causing pressure on the pulp; then apply oil of cloves on a pledget of cotton the size of a pinhead and cover with dry cotton. Fill over this. Oxide of zinc and oil of cloves make an anodyne and antiseptic paste which may be flowed over an exposed pulp and protected by a filling of gutta-percha or cement. The pulp will probably die easily under this, and after a week or two the canals can usually be cleansed and filled. If abscessed they should be cleansed mechanically and packed with cotton saturated with oil of cloves, which, by means of pressure with unvulcanized rubber placed in the cavity, should be forced out through the fistulous opening. The pulp chamber may then be flooded with a solution of gutta-percha in eucalyptol and some temporary stopping forced into each canal until the eucalyptol shows at the opening of the fistula. They will rarely give further trouble.

The first permanent molars are called upon to do longer service than any other tooth in the mouth, and have a very important function during the growth of the bicuspids and second molars to full length. If not kept in place the jaws are allowed to drop together so that the upper incisors overlap the lower more than normal, and the bicuspids and molars never acquire their full length. Every effort should, therefore, be made to preserve the first molars. On the slightest approach of caries they should be carefully filled. If they are much broken, build them up, or crown them if necessary. The material to be used must be governed by the ability of the patient, or the disposition to withstand dental operations.

It is well to use cement on the occlusal surface as a prevention, forcing it into the grooves and sulci, renewing it until conditions make it possible to insert metal fillings—"conditions" here referring to expediency and forbearance on the part of the patient, rather than to any pronounced change in structure. The choice between gold and amalgam depends upon the question of expense and the ability of the patient to submit to gold operations. It is a question of physical and mental stamina on the part of the patient. The mesial surface of the first molar calls for the most careful attention, as it is in contact with the deciduous molar, and if the latter is affected on the distal surface the former is almost certain to suffer. It is well in many cases to grind away the distal surface of the deciduous molar. If decay occurs control it with gutta-percha or cement until the deciduous molar is lost, when the permanent molar should be promptly and permanently filled with gold while it is fully exposed. If decay occurs early in the permanent incisors it should be controlled with gutta-percha or cement until the patient is schooled into an attitude of sufficient forbearance to submit to gold operations, as early as may be practicable.

As the mineral becomes the vegetable and the vegetable becomes the animal through the action of vital energy, rising a step higher—from mineral to mind—there is no break. Through it all there is a vital principle that can not be weighed, can not be measured. A tooth is developed which corresponds with another on the opposite side of the mouth in size and form and color that is divine, and so it is with all the other organs. The tooth is sustained by vital energy; when it becomes inert it is cast out. Taste is vital electricity. When silver is worn in the mouth, coming into contact with food the current is converted into organized electricity and gives an unpleasant taste. Metal in the dentin which comes in contact with the pulp also creates a current which is connected with galvanized electricity. Food produces a vital current and electricity carries on the work.

Dr. Corydon Palmer said, the older men present had all been familiar with the use of tin and gold. He had used it himself as far back as 1839. If it was good then, it is good now. It is better than anything except pure gold, and for many cases it is even better than gold. With teeth of good structure, and with all other conditions favorable, gold was undoubtedly the best material, but there are cases which require cement, and gutta-percha is best for others. There is no subject of greater interest than the management of children's teeth. Begin early with the little children; watch them carefully while they are changing their teeth and getting their sixth-year molars. If there is a little decay, fill them promptly. Do not use tin and gold simply because it is easy to use it in that way, you can do it quickly and make a nice operation; do not use cement or amalgam because you can let the patient go quickly and make room for another; but do what is best for the tooth in each case. But do not subject the little ones to too severe operations. There is nothing better than tin cut in strips and folded over, of the right width and made into cylinders.

DISCUSSION.

Dr. B. Holly Smith: The conservative spirit of Dr. Johnson's paper appeals forcibly to me. The day has come when old-fashioned dentistry has to go. Children will cease to come to us crying and afraid; they will no longer approach us with dread. The youngsters will come to us willingly and gladly. I congratulate the modern conservative dental operator on the new attitude of the public mind toward dentistry. It has been said that the dental profession is increasing out of proportion to the population. But with dental practice as humane and conservative as that advocated by Dr. Johnson, there will be fifty percent more done. People will no longer stay away from the dental office because of wrong impressions and dread of dental operations.

Dr. Garrett Newkirk: It is often quite difficult to keep the teeth of little children dry; you can not always use the rubber-dam or clamps. I have often found it advantageous to make the little patient my assistant. Give them a hand-glass and adjust an absorbent pad or a piece of spunk in place, and show them how to hold it between the cheek and the tooth. They will often be so greatly interested in being of assistance in this way as to forget their discomfort.

Dr. Darby: I rise not to criticise but to commend, and also to put in an interrogation mark. Ought arsenic ever to be used in deciduous teeth? It has been spoken against most positively. If I had been asked the question ten or fifteen years ago I should have said it would be positively reprehensible; that it would be bad practice; that creosote and cantharides were all that were required. But my views on this question have changed. I know of no reason why it should not be used with proper precautions, and in the last ten years I have so used it in very minute quantity, and I have yet to see the first indication of bad results. Another point, and that is as to the use of gold in the permanent teeth of young children. Just as soon as it is desirable, or the patient is prepared to stand the wear and tear of the gold operation, it is said to be the better practice that we should make permanent operations as early as possible. But the pulp must always be taken into consideration, and the dangers from thermal changes. It is better to hold them along with cement and gutta-percha. Not until, I do not dare to say in the presence of Dr. Black until the teeth have attained a greater degree of hardness, but until the teeth have matured. I must give some credit to the results of experience and observation, though I am less ready now than formerly to put theory against observation. I know that Dr. Black's science is worth more than my observation. Formerly I would have said that great changes do take place in the structure of the teeth—changes from good to bad, or from bad to good. At the age of twelve I should have said this was my position; at the age of thirty-five or forty, less certain. But I am afraid to put my observation against the scientific knowledge of such men as Dr. Black and Dr. Williams. But I must disagree as to the use of gold in young teeth.

Dr. H. J. McKellops: I must take exception to some points in this paper. Young teeth can be filled with gold, but I have urged ever since the cements came in that by the use of oxy-phosphate we can keep the teeth along to the age when we can operate as we desire to do. But even then a foundation of cement at the bottom will save two-thirds of the work. There will be no decay under it, and you can put what you please on top of it. Pay attention to these little things, and you will get fine results and save much suffering. For old people who can not stand much you can save the teeth for years and years, when they can not stand the insertion of other materials. I know of one lady who will have nothing but cement in her mouth. She travels a great deal, goes everywhere, and has no trouble with her teeth. If it wears out, it can be replaced with but little trouble. I am not ashamed to have any one look at my work of this character. Try it and see what you can accomplish with it. If you will use amalgam, put cement under it, and keep the mercury away from the dentin. It will create no soreness and never leaks.

Dr. A. W. Harlan: I am astonished at the position taken by one of the essayists on the penetrability of cement, as shown by cylinders placed in bottles, which were all permeated with the dye. He said that no cement was sold to dentists that would not either expand or contract; that cement was not a reliable filling material. And yet this evening Dr. McKellops, an old practitioner, and Dr. Darby, a professor of operative dentistry, say that the cements will and do preserve the teeth. So that laboratory experiments and practical experience do not go hand in hand. What are we to do? Shall we go on using the cements, or shall we abandon them? If the experiments are to be relied upon, it is not safe to use them in the teeth, because they leak. But the conditions in the laboratory experiments are not those which exist in the mouth. Some manipulators, too, are so skillful that they always mix their cements so that they do not either shrink or expand, though it is also true that there are many who can not so mix them. Dr. Darby says he uses arsenic for destroying pulps in deciduous teeth. But even his experience in this direction is not a safe guide; the danger is too great in consequence of the uncertainty of retaining the application, especially in the presence of the fact that all known cements leak. A well-known writer in the *Cosmos* has recently advocated the use of arsenic for sensitive dentin, but I apprehend that sooner or later he will find those teeth pulpless if not abscessed. The presence of arsenic is fatal to the vitality of the pulp. The non-appearance of the bicuspids is due to the use of arsenic in deciduous molars, the escape of arsenic destroying the germ of the permanent tooth. This does not follow in all cases, but undoubtedly has in more than one that arsenic has produced necrosis. It is not safe for the vast majority of practitioners to use such a corrosive agent to destroy the pulp of a deciduous tooth. There are other means of effecting this without the risk of injury to the germ of the permanent tooth. We are often restricted in our methods by the lack of physical strength or mental stamina in children. When we are not able, for this reason, to do the thorough work we would like to do, it is better to sterilize the pulp chamber and leave the root canals open, even resorting to the old-fashioned plan of drilling into the pulp chamber beneath the gum rather than attempt ineffectual root filling.

Dr. Sitherwood: There is nothing in the whole range of practice that gives more trouble than the root canals of deciduous teeth, but for the past year I have filled a large number practically as Dr. Johnson has described. I am in accord with Dr. Johnson as to the use of cement, but the manipulation in mixing and also the temperature has a great deal to do with success or failure. I have been gratified with what Dr. Black has said relative to immunity in the deciduous teeth. We often find that decay has progressed to a certain extent and then has ceased. We find the cavity has taken on a brown color and decays no further. As to the use of nitrate of silver, I have found that the citrate of silver is much better; there is nothing so useful in the deciduous teeth.

Dr. George Vann: A number of years ago Dr. Wm. G. A. Bonwill demonstrated in the city of Atlanta, his method of using gutta-percha in proximal cavities in children's teeth, not merely to preserve the teeth, but to preserve the space in the jaw, inducing development of the jaw for the accommodation of the permanent teeth. I had some conversation with Dr. Bonwill on the subject, and on my return home began that practice and the results have been most satisfactory. Dry out the cavities and apply a solution of balsam dissolved in chloroform, and then the gutta-percha. It is done very quickly and is easy to the little patient, and space is insured for the advent of the permanent teeth.

Dr. Ames: I would like to correct the impression that there is a discrepancy between the results with cement in the teeth and in the laboratory. No claim has been made that every cement will shrink or leak in the mouth. It is only in the dry condition from lack of water of crystallization. In the mouth, in contact with dentin, shrinkage is overcome, because it is kept moist at all times. There are different degrees of porosity in the cements, but I think it is possible to make a cement that will not be porous. Dr. Johnson presses the cement in hard with the finger. Dr. Pearsall advocates the use of a tin matrix that can be pressed against the cement, and makes a beautiful surface. I would like some one to try chloride of gold crystals, slightly liquefied and applied to the surface of a cavity, as nitrate of silver is used.

Dr. Wm. Conrad: By the applause that greeted words spoken in favor of amalgam, one would judge that this society was becoming a body of amalgam-stuffers. Many are going back to sponge gold. I am sorry to see such applause given to amalgam, for it has done more harm to the dental profession than all other causes put together. A low grade of dental practice makes a market for amalgam. I have used arsenic for twenty years and never had but one serious case. Then I stuffed arsenic into the roots of a tooth, and lost the tooth as I deserved to do. But in twenty-two years I have had only that one lame tooth.

Dr. G. V. Black: I have limited the easy filling of children's teeth as closely as it should be limited. We must not destroy the confidence of the child, nor break down its courage, nor injure the nervous system. But to do our whole duty, we should fill permanently at the earliest possible moment, and there will not be so much to do in later life. I detest the idea of temporizing, and yet in many cases we are obliged to temporize. We should not put in a big gold filling independent of conditions, but we can endeavor to bring about the conditions that will permit the insertion of the gold filling. I have put in many for children eight years old, and I have not found that the pulps died in consequence. Pulps do not die under gold fillings in children's teeth in as great proportion as in the teeth of adults. As to the conditions of immunity, there is a whole lot we don't know yet, but we should begin to study the question earnestly and endeavor to elucidate it. It will require much time, and much thought, but by putting together a suggestion from one man, and a thought from another, we shall eventually reach something positive and definite. The fact is admitted that conditions of immunity are confronting us continually. It is only those who have something the matter with their teeth that come to us. Many go through life without decay; with nothing wrong; consequently they do not come to us—such are to be found in every community. Let us seek out such cases, and examine and study them. Many have diseases of the peridental membrane who have no decay. Others attain a condition of immunity after violent ravages of decay. I wish the dental profession would take up the study of the condition of immunity. It is not a subject for laboratory study and investigation. In this line clinical study must precede laboratory study. Take it home with you as a subject of thought and report your findings.

